# Enhancing the Tracking of Seedling Growth Using RGB-Depth Fusion and Deep Learning

**DOI:** 10.3390/s21248425

**Published:** 2021-12-17

**Authors:** Hadhami Garbouge, Pejman Rasti, David Rousseau

**Affiliations:** 1 Université d’Angers, Laboratoire Angevin de Recherche en Ingénierie des Systèmes (LARIS), UMR INRAE IRHS, 62 Avenue Notre Dame du Lac, 49000 Angers, France; hadhami.garbouge@univ-angers.fr (H.G.); pejman.rasti@univ-angers.fr (P.R.); 2 Centre d’Etudes et de Recherche pour l’Aide à la Décision (CERADE), École D’ingénieur Informatique et Environnement (ESAIP), 49124 Angers, France

**Keywords:** deep learning, plant growth, CNN, RGB-Depth, image fusion, feature fusion, transformers

## Abstract

The use of high-throughput phenotyping with imaging and machine learning to monitor seedling growth is a tough yet intriguing subject in plant research. This has been recently addressed with low-cost RGB imaging sensors and deep learning during day time. RGB-Depth imaging devices are also accessible at low-cost and this opens opportunities to extend the monitoring of seedling during days and nights. In this article, we investigate the added value to fuse RGB imaging with depth imaging for this task of seedling growth stage monitoring. We propose a deep learning architecture along with RGB-Depth fusion to categorize the three first stages of seedling growth. Results show an average performance improvement of 5% correct recognition rate by comparison with the sole use of RGB images during the day. The best performances are obtained with the early fusion of RGB and Depth. Also, Depth is shown to enable the detection of growth stage in the absence of the light.

## 1. Introduction

The detection of the seedling growth stages is a fundamental problem in plant science. This covers the emergence of seedling from the soil, the opening of cotyledons and appearance of the first leave which correspond to the earliest stages of development of plant. The success or failure of these developmental stages and their kinetics have a huge impact on the evolution of the future plant. Recently, seedling growth monitoring has received attention from the computer vision community [1,2,3,4,5,6,7,8,9,10,11,12,13,14,15,16]. Among these works, the state-of-the-art approach based on deep learning proposed in [16] has shown the possibility to automatically classify the stages of development of seedling with RGB sequences of images from top view with an accuracy higher than 90%.

One of the limitations of the work proposed in [16] is that the monitoring was done only during daylight with RGB images. Consequently, any events happening during the night would be missed and/or possibly estimated with a temporal bias. In this article, we propose an extension of the work of [16] and investigate the possibility to push forward the monitoring of the seedling growth during the day and the night. To this purpose, RGB-Depth camera were used. These technologies have been demonstrated of wide value in plant phenotyping [17,18,19,20,21,22,23,24]. The depth images are computed by an active LIDAR camera operating in infrared (IR). This camera can be activated during day and night without impact on the development of the plants. As in [16] we selected low-cost versions of these RGB-Depth cameras. These low-cost constraints are specially important in plant phenotyping [25] when moving the plants or the camera is not an option and that replication of cohorts of cameras is to be chosen to monitor large populations of plants. Low-cost RGB-Depth cameras are also logically coming with artifacts and noise. Such artifacts and metrological limitations of low-cost RGB-Depth cameras have been extensively studied (see [26] for a recent survey). In our case, we rather work at an informational level. We focus on a classification task, i.e., a nonlinear decision, which is by nature more robust to noise since it does not have to provide a high-fidelity, metrological, linear estimation. The hypothesis investigated in this article is that these low-cost RGB-Depth sensors despite their limited spatial resolution and the presence of artifacts may be of enough value to enhance the tracking of seedling growth during day and night.

We demonstrate, for the first time, to the best of our knowledge the value of these RGB-Depth images to monitor the early stages of seedling growth. We investigate fusion strategies between RGB and depth with several neural networks architecture. The underlying motivation to use multimodal data is that complementary information give a richer representation that may be utilized to create better results than a single modality. The multimodal fusion research community has made significant progress in the past decade [27]. Different fusion strategies have been reviewed [28,29]. Specifically for RGB and Depth with deep learning architectures, fusion has been extensively studied in the literature [30,31,32,33,34,35,36,37,38,39,40,41]. Mainly two types of fusion can be distinguished. First, images can be stacked at the input: this is the early fusion [30,31,32,33,34], that we call image fusion. Second, deep features can be independently extracted and then fused before a classification stage: this is the feature fusion [35,36,37,38]. In this work, we investigate these fusions scenarios that we applied to the important problem of seedling growth stage monitoring. Since we process sequences of images we considered time-dependent neural network architectures. As in [16], we included a base line convolutional neural network (CNN) and LSTM [42]. We also added TD-CNN GRU [43] and transformer [44] which were not included in [16].

## 2. Materials and Methods

### 2.1. Imaging System and Data Set

**Figure 1:**
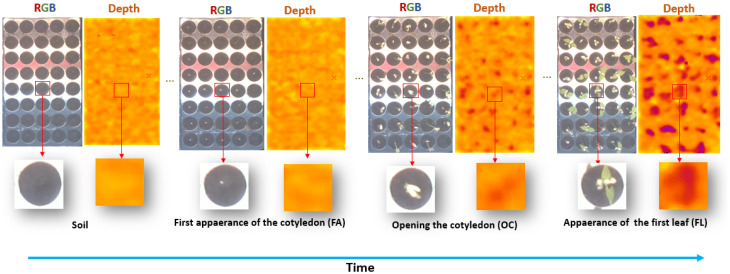
Overview of the time-lapse collected for this work. Upper row, view of a full tray with 72 pots from top view. Lower row, a zoom on a single pot at each stage of development to be detected from left to right: soil, first appearance of the cotyledon (FA), opening the cotyledons (OC) and appearance of the first leaf (FL).

We have conducted similar experiments as the ones described in detail in [16] and shortly recalled here. A set of minicomputers, connected to RGB-Depth cameras [45], was used to image seedlings from the top view as illustrated in Figure 1. We used, instead of the RGB cameras of [16], Intel real sense cameras [46] (model D435) which natively produces registered RGB-Depth pairs of images and calibrated Depth maps. We installed 8 of these RGB-Depth cameras in a growth chamber where cameras followed the growth of seedlings from top view. During experiment, soil pots were hydrated to saturation for 24 h after which excess water was removed. After 24 h, seeds were sown at a depth of 2 cm, and trays were placed in a growth chamber at 20 °C/16 °C, with 16 h for photoperiod at 200 μMm${}^{-2}$ s${}^{-2}$. The soil was kept wet throughout the experiments. Each experiment took one week with a frame rate of 15 min. The time lapse program (made in Python) was implemented on a central minicomputer controlling, via ethernet wires, the 8 minicomputers connected to the RGB-Depth cameras.

Concerning the biological material, seedling growth was recorded for 2 experiments using seed lots from different accessions of beans such as Flavert, Red Hawk, Linex, Caprice, Deezer and Vanilla. Each experiment consisted of 3 trays with 40 pots in which 120 seeds of accessions were sown. There is a similarity between the species in this experiment and the two species which were used in [16] as all of them consist in dicotyledon species. The main difference between them comes from the number of varieties in this experiment which is three times higher than the one in [16].

In total, the database consists of 72 temporal sequences of RGB and depth images of size 66×66 pixels where each temporal sequence consists of 616 individual images. Example of images from the database is shown in Figure 1. RGB-Depth temporal sequences acquired during daylight were annotated by expert in biology while looking at RGB images. This ground-truth annotation consisted of four classes: soil, first appearance of the cotyledon (FA), opening of the cotyledon (OC), and appearance of the first leaf (FL). The algorithms presented in this paper for seedling emergence identification following these four phases of growth were trained, validated, and tested against this human-annotated ground-truth. In order to train robust models, we used the cross-validation approach by considering image sequences of bean varieties in three split of train, validation, and test dataset. Table 1 provides a synthetic view of the data set used for training and testing of the models. For the training dataset, we applied data augmentation using a simple horizontal flip on each temporal sequence.

Depth images can contain artifacts with missing values. This can happen on part of the scene where not enough light is reflected or for objects that are too close or too far from the camera. While neural networks should be able to cope with such noise, it is better to correct them to use the capability of these networks on clean data. In order to correct these artifacts, we applied a classical inpainting technique [47] of depth images to reduce the noise.

### 2.2. RGB-Depth Deep Learning Fusion Strategies

**Figure 2:**
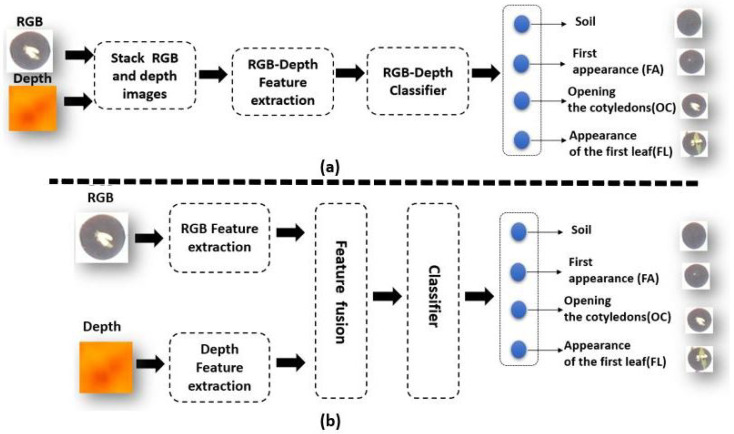
Different types of RGB-Depth fusion architectures tested in this article for image classification. (**a**) Image-based RGB-Depth fusion, (**b**) Feature-based RGB-Depth fusion.

We describe here the different neural network architectures tested in this study to fuse the RGB and Depth for the classification of seedling growth stages as depicted in Figure 2.

#### 2.2.1. CNN-Based Image Early Fusion Learning Structure

**Figure 3:**
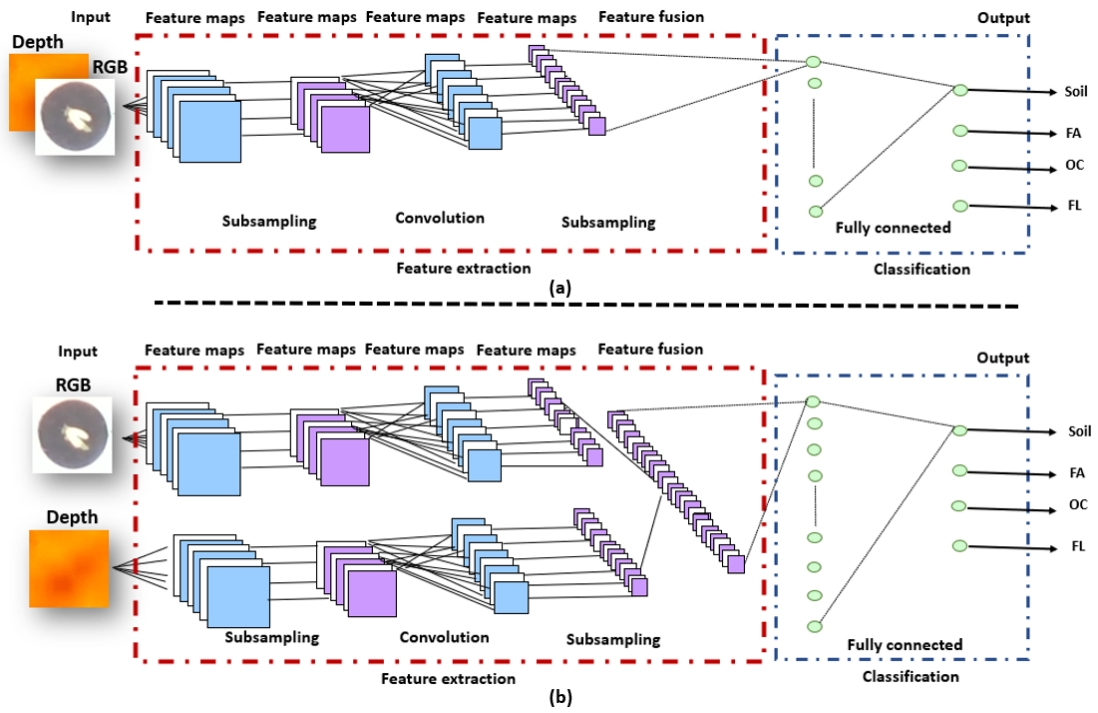
(**a**) CNN architecture of image fusion for RGB-Depth, (**b**) CNN architecture of features fusion for RGB-Depth.

We first integrated, as in [48], RGB and Depth data stacked in a four-channel as input to a CNN (see Figure 3a). The feature extraction block from four-channel input images is followed by the classification block (shown in Figure 3a). The CNN architecture is the one of [16,43] that we shortly recall. The feature extraction block of a CNN model is responsible for extracting features from input images using convolutional layers, whereas the classification block determines classes. To keep the amount of train parameters low, we created an AlexNet [49] like CNN structure. This architecture reads as follows: four convolutional layers with filters of size 3×3 and respective numbers of filters 64, 128, 256, and 256 each followed by rectified linear unit (RelU) activations and 2×2 max-pooling; a fully connected layer with 512 units, ReLU activation and dropout (*p* = 0.5) and a fully connected output layer for four classes corresponding to each event with a softmax activation. This proposed CNN architecture has been optimized on a hold-out set and was demonstrated in [16] to be optimal by comparison with other standard classical architectures (VGG16, ResNet, DenseNet). The network was trained from scratch since the size of the input tensor (4 channels and small spatial resolution) was different from existing pre-trained networks on large RGB data sets.

#### 2.2.2. CNN-Based Feature Fusion Learning Structure

Our architecture, shown in Figure 3b, is made up of two convolutional network streams that operate on RGB and Depth data, respectively. The same structure of image fusion CNN has been developed for each stream of the feature fusion CNN. The feature extractor part of the CNN architectures of RGB and Depth images consists of four convolutional layers which have 64, 128, 256, and 256 filters, respectively (similar to the AlexNet like structure of the previous subsection). The ReLU activation function is considered for each convolutional layer followed by a max-pooling layer. On the classification part of the CNN architectures, a fully connected layer with 512 units, and an output layer with four neurons corresponding to each event with a softmax activation function.

#### 2.2.3. TD-CNN-GRU-Based Image and Feature Fusion Learning Structure

**Figure 4:**
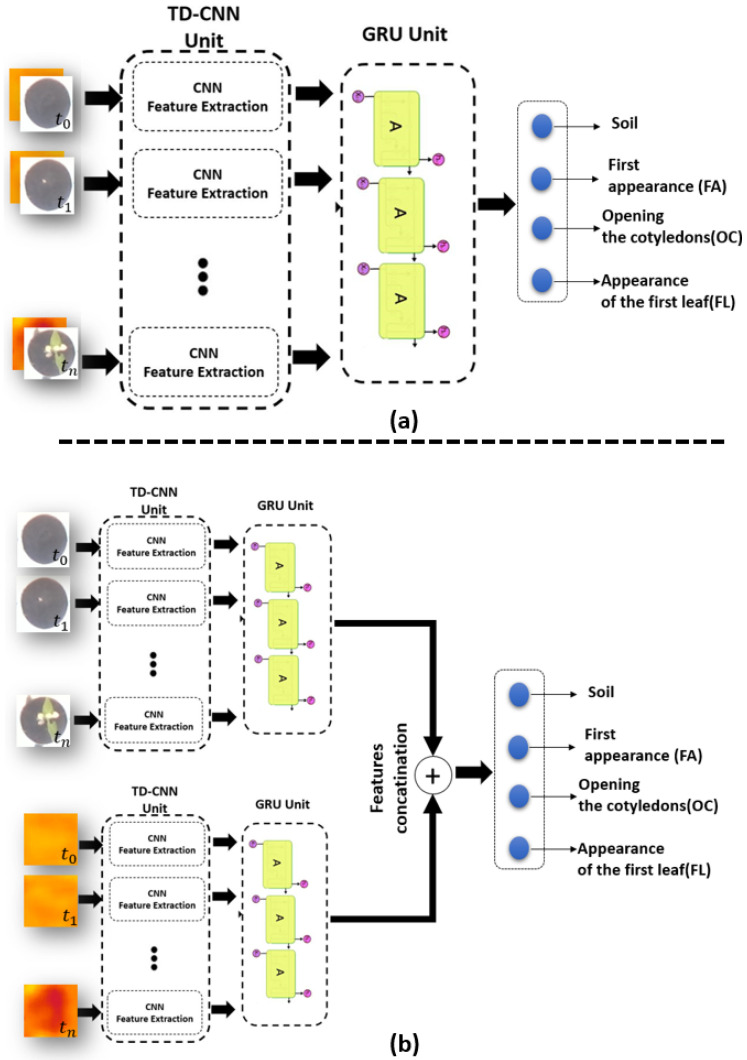
(**a**) TD-CNN-GRU architecture of image fusion for RGB-Depth, (**b**) TD-CNN-GRU architecture of features fusion for RGB-Depth.

We demonstrated in [16,43] the possible added value to embed in controlled environment a memory in the process of the sequence of images. We demonstrated in [43], the superiority of Time dependent CNN with gated recurrent units (TD-CNN-GRU) by comparison with other memory based methods such as long short term memory (LSTM) and CNN-LSTM architectures. GRU uses two gates: the update gate and the reset gate while there are three gates in LSTM. This difference makes GRU faster to train and with better performance than LSTMs on less training data [50]. The same CNN architecture of our model in [16] was embedded in our TD-CNN-GRU model where the optimal duration of the memory was found to be 4 images in [16,43] corresponding to 1 hour of recording. Figure 4 shows a schematic view of the proposed TD-CNN-GRU for images and feature fusion respectively.

#### 2.2.4. Transformers-Based Image and Feature Fusion Learning Structure

**Figure 5:**
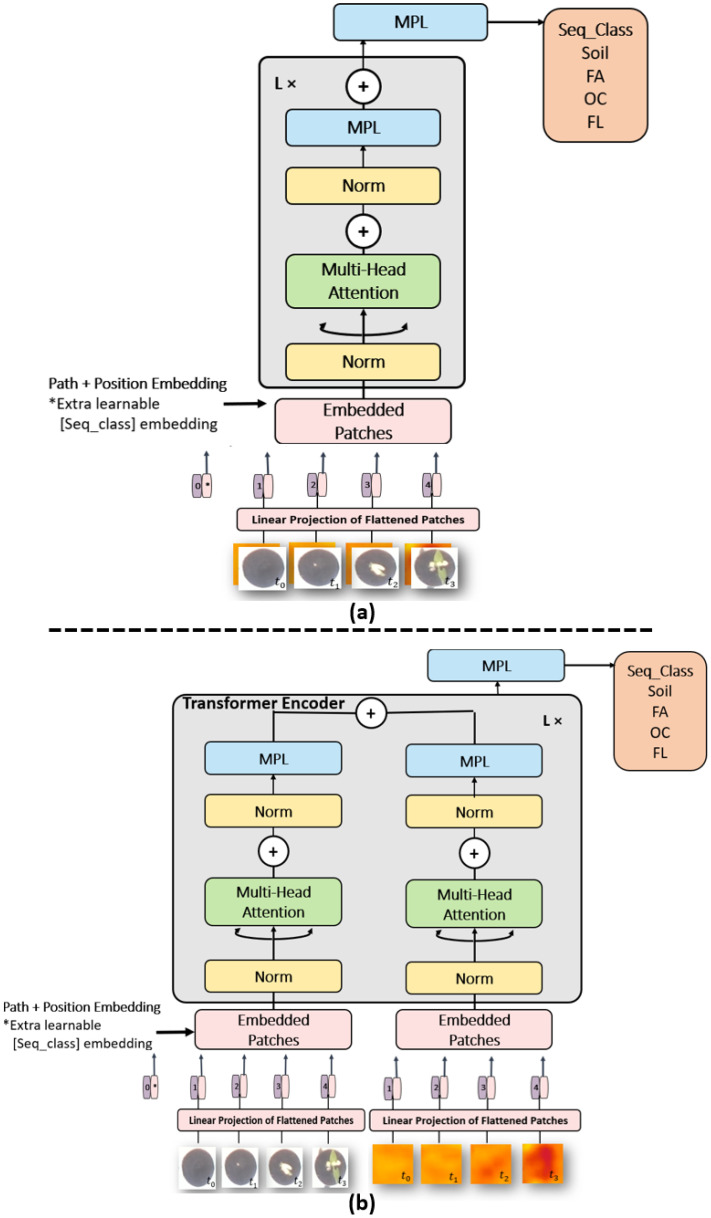
(**a**) Transformer architecture of image fusion for RGB-Depth, (**b**) Transformer architecture of features fusion for RGB-Depth.

A last class of neural network dedicated to time series are the transformers. Since their introduction in [44] they have been shown to outperform recurrent neural networks such as LSTM and GRU specially in the field of natural language processing as they do not require that the sequential data be processed in order. Transformers have been shown suitable to process temporal information carried by single pixels in satellite images time series [51,52,53]. Transformers have recently been extended to the process of images [54] where images were analysed as a mosaic of subparts of the original images creating artificial time series. In our case, we directly have meaningful original images which corresponds to the field of view of the pots. We, therefore, provide the transformer of [54] with time series of consecutive images of the same pot (we used the same time slot as in the other spatio-temporal methods). We used 32 transformer layers with batch size 64, feed forward layer as classification head layer and the size of our patch size was equal to 66×66 pixels for both architectures of Figure 5.

For all our training, we used the NVDIA DGX station. This station is composed of 4 GPUs and each one of them have a RAM memory of 32 Gb. We used Python version 3.7.8, Tensor-flow version 2.7.0 and Keras library version 2.3.1.

### 2.3. Accuracy

The performances of the different fusion strategies tested on our dataset were classically assessed with *Accuracy*

(1) $$Accuracy=\frac{TP+TN}{TP+TN+FP+FN}\;,$$

where *TP*, *TN*, *FP*, and *FN* stands for true positive, true negative, false positive, and false negative).

## 3. Results

### 3.1. Fusion Strategies

**Figure 6:**
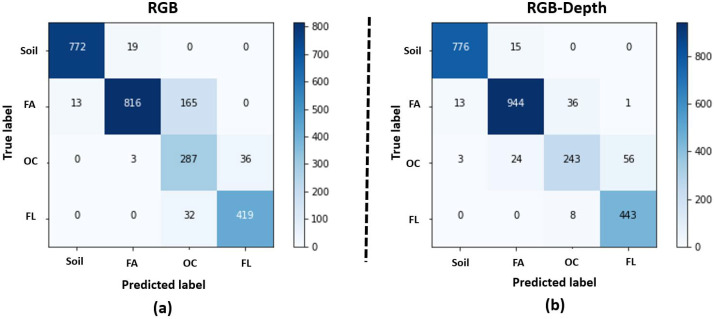
Confusion matrices for the best method found in Table 2, i.e., CNN. (**a**) for the RGB images and (**b**) for the RGB-Depth images.

The proposed deep learning methods CNN, TD-CNN-GRU, and Transformers with image or feature RGB-Depth fusion were applied to the produced dataset as described in the Section 2. The performances are provided in Table 2, Table 3 and Table 4 and Figure 6.

Table 2, Table 3 and Table 4 show that all methods performed better when RGB and Depth data are fused by comparison with the sole use of RGB data. This improvement is obtained both with image fusion and with feature fusion. This demonstrate the value of RGB-Depth fusion with a gain of 5% (on average) compared to the use of the sole RGB images. This is obtained at a reasonable training time of around 1 to 3 h as detailed in Table 5. The best results are obtained with the CNN method, i.e., the spatial method by comparison with the spatio-temporal method. This CNN is showing the best absolute performance, the smallest training time and also minimum decrease of performance between training, validation and test. This is in agreement with our previous results found in [16,43], where spatio-temporal methods outperformed memoryless spatial ones only when the kinetic of growth were homogeneous among the dataset. This was not the case in this study.

The confusion matrix of the CNN method is displayed in Figure 6 for RGB images and RGB-Depth images. Interestingly errors with both RGB and RGB-Depth only occur on adjacent classes along the developmental order. These are situations where even the human eye can have uncertainty to decide the exact time of switching from one class to the next one. Remaining errors can thus be considered as reasonable errors. The confusion matrices also clearly demonstrate that the main gain brought by the Depth channel is on the stage of opening the cotyledons for which the error are divided by a factor two. First appearance out of the soil, or the appearance of the first leave produce very limited variations on the depth. By contrast, the opening of the cotyledons produces an abrupt variation of the Depth. Therefore, the impact of Depth on the improvement of the performance of classification on this developmental stage is consistent with this rationale. Following also this rationale, one can notice that the errors on opening the cotyledon slightly increase when Depth is added but the overall impact of Depth is on average beneficial to the global accuracy.

### 3.2. Detection of Event Changes at Night Using Depth Information

**Figure 7:**
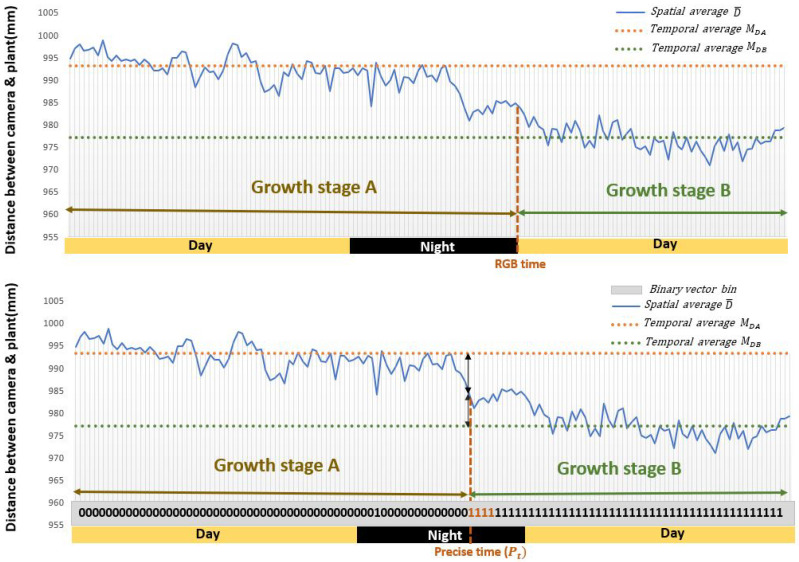
First row: the detection of switch from growth stage A to growth stage B using only daytime RGB images. Second row: the more precise detection of switch from growth stage A to growth stage B using the Depth pattern during the night time as proposed by Algorithm 1.

The advantage of using the depth is not limited to enhance the performance during the day as shown in the previous subsection. Depth is also expected to be specifically useful during the night since the RGB cameras are then non operating while the Depth images can still be acquired. If the growth stage switches during the night the RGB imaging devices detect the switch only on the first frame of the next day time as illustrated in Figure 7. It is possible to screen for Depth alone during these nights and observe the start of a growth pattern actually occurring before the beginning of the day. We demonstrate in this subsection how to take benefit quantitatively of the sole Depth channel during these nights.

**Figure 8:**
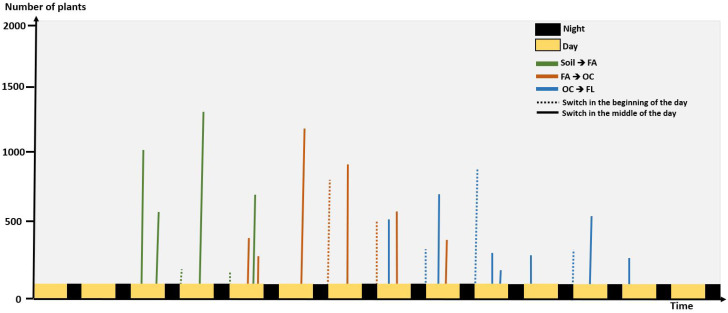
Histogram of detection of growth stage change during day and night from 4000 plants.

We analyzed the number of switches from one growth stage to another happening on the first image acquired during the day in the data set of [16] and found out that it represented 35 percent of the events (see Figure 8). This is similar to what we found with the dataset of this article where we had 100 sets of pots from different varieties. In these frames, we have 115 switches of growth stages with 43 happening during night time. While some could be triggered by the action of light others could also happen earlier during the night. To detect a possible change during the night, we quantitatively used Depth. We designed Algorithm 1 which acts as follows. We first detects nights where a switch between a growth stage to another growth stage is found in RGB images. During these nights, the algorithm then detects the depth frame on which the switch is the most likely to occur. In short, this is obtained by choosing the time where the average spatial depth is permanently (computed over a sliding window of 4 images = 1 h) closer to the average spatial depth of the next growth stage.
**Algorithm 1:** Detection of night events using depth information.
**Input**:
$S^{night}$ = Sequences of depth images of a night during which a switch a growth stage is observed in RGB images.
$S^{a}$ = Sequences of depth images from the last day before the switch of growth stage A to B.
$S^{b}$ = Sequences of depth images from the first day after the switch of growth stage A to B.
**Output**: $P_{t}$ = Precise time of switch of growth stage.**1**$\bar{\mathrm{DA}}$← mean($S^{a}$);▹ Spatial average of $S^{a}$**2**$\bar{\mathrm{DB}}$← mean($S^{b}$); ▹ Spatial average of $S^{b}$**3**$\bar{{\mathrm{DN}}_{k}}$← mean($S^{night}$);▹ Spatial average of $S^{night}$**4**$\langle M_{DA}\rangle$← mean( $\bar{\mathrm{DA}})$;▹ Temporal average of $\bar{\mathrm{DA}}$**5**$\langle M_{DB}\rangle$← mean( $\bar{\mathrm{DB}})$;▹ Temporal average of $\bar{\mathrm{DB}}$**6**GA←$\bar{\mathrm{DN}}$ − $\langle M_{DA}\rangle$;▹ Difference between $\bar{\mathrm{DN}}$ and $\langle M_{DA}\rangle$**7**GB←$\bar{\mathrm{DN}}$ − $\langle M_{DB}\rangle$; ▹ Difference between $\bar{\mathrm{DN}}$ and $\langle M_{DB}\rangle$**8**bin← sign (GA − GB); ▹ Binary vector of the sign for the difference between GA and GB**9**Idx← find(*bin*==1111);▹ Get the index of first pattern (1111) in the binary vector.**10**$P_{t}$←$Length(S^{a})$ + Idx; ▹ Add the length of $S^{a}$ to the index of the first pattern (1111) to get the precise time

To validate Algorithm 1, we could not establish ground truth during the night. As a workaround, we used daylight events and applied the depth channel only to the Algorithm 1. Then, we used the annotated ground truth obtained from the RGB images to compute the performance of Algorithm 1. We found 80% of these 115 switches with a shift of less than 4 frames on average (standard deviation of 2 frames) by comparison with the manually annotated ground truth. This corresponds to an uncertainty (bias here) of 1 h which is very reasonable and much lower than the error duration of the night itself (8 h) if no Depth were used.

## 4. Discussion

We analyzed the remaining errors of the proposed algorithms and discuss them in this section together with some open perspectives of the work.

**Figure 9:**
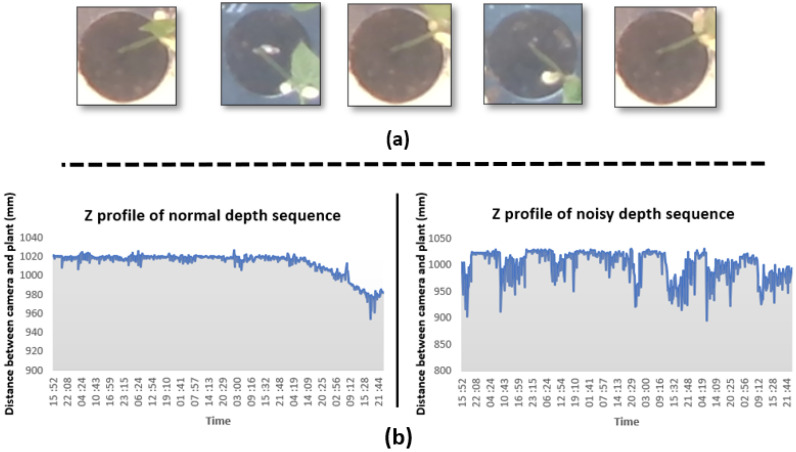
Sources of errors due to the acquisition protocol (**a**) and instrumentation (**b**).

**Figure 10:**
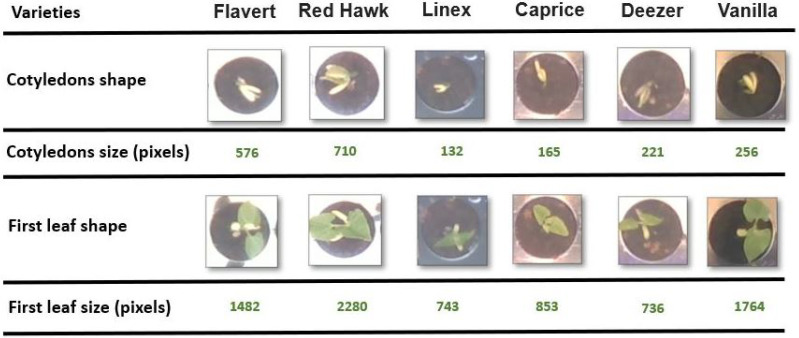
Heterogeneity of shape and size in the two events OC and FL for the different bean varieties used in the training.

Two main sources of errors can be attached to the acquisition protocol and instrumentation itself. These are illustrated in Figure 9. First, some seedlings growth so fast that their leaves or cotyledons go out of the observation window (Figure 9a). This causes drop in depth and change in the RGB pattern. With our current approach, we do focus on individual pots. For such seedlings growing at early stages outside of their pot, we would need to either use larger pots or develop tracking algorithms. This falls outside of the scope of this study which focused on the added value of Depth when fused to RGB for the detection of early growth stages of seedlings. Another source of errors happens due to noise on the Depth channel (Figure 9b). Such noises were observed when too much or too low amount of IR light was reflected on pots. This happens for instance when the plastic material of the pots has a high reflectance or when some remaining water(absorbing IR) is present. These noises can be reduced by carefully choosing the material used for the pot and the watering process. Another type of error comes from the inherent large heterogeneity of shapes and sizes of the bean varieties considered in this study and illustrated in Figure 10. This affects specially the detection of growth stage which shows the tiniest changes, i.e., the opening of the cotyledons. To solve these errors, one could simply add more data or use more advanced data augmentation techniques such as zoom, stretch, color jitter, …We wanted to provide basic results here which already happen to be of rather high quality without the use of such approach to robustify the model since the main goal was the fusion of the RGB and Depth for seedling growth monitoring.

One may wonder about the robustness of the model proposed given the relatively small size of the plant population considered. First, the overfit measured with the best method was found to be limited together with the difference of performance between cultivars. It is important to recall here that the point of the work is to quantify the added value of RGB-Depth images by comparison with sole RGB. This is what we do on the same data sets. Interestingly, the performance with RGB images obtained with only 72 samples are similar to the larger data set used in [16] (90% against 88% here). However, we cannot ensure a perfect robustness to large change of phenotypic shapes. If such variability in scale was expected, larger data sets would have to be constituted. The comparison between RGB and RGB-Depth would remain unchanged.

In this work, we focused on early fusion and feature fusion of RGB and Depth. One may also consider decision fusion where the classification from the RGB image and the Depth image would be made. We performed this analysis and found a pure random decision when the classification was made on Depth alone. Therefore, at the decision level, no added value of Depth was to be expected on average. Fusion between RGB and Depth for such small images and low-cost sensors as the one considered in this study is found to be beneficial on average at earlier stages of processing (image or features). However, after analysing the confusion matrix in detail, one could imagine to selectively using the added value of Depth at the stages of growth where it is expected to be the most significant. This was found to be between the FA and OC in our case and more generally when large contrast in Depth happens. On the contrary, one could discard the use of Depth when the growth process is estimated to lay at stages where no contrast in Depth is expected (between Soil and FA in our case).

This work could be developed in several other future directions. First, we could revisit this study with higher resolution Depth sensors [26] to investigate how the reduction of noise and improvement of resolution in Depth could help to further improve the classification results. More advanced stages of development yet still accessible from the top view, could be investigated without targeting 3D reconstruction [55]. An issue comes with the possible overlapping between plants. One solution would be to decrease the density of plants but this would come with a lower throughput for the experiments. Another solution would be to investigate the possibility to track leaves during their growth in order to decipher partial occlusions. Here again, RGB depth sensors coupled with advanced machine learning approaches could be tested to further extend the capability to monitor seedling growth [56]. Last but not least, we can now directly apply the developed algorithms to analyze biologically in detail the statistical distribution of seedling growth events at night on large datasets. This may unravel new knowledge on the physiological impact of light on these growth kinetics in addition to their links with circadian rhythms [57].

## 5. Conclusions

In this article, we have demonstrated the added value of Depth when fused with RGB images for the important problem of detection of seedling growth stage development. During day time, Depth was shown to improve by 5% the classification performances on average. Also Depth was shown of value to refine the estimation of switch of growth stage during the night period. These results were established on different fusion strategies including CNN, TD-CNN-GRU and transformers. These methods were compared in order to incorporate the prior information of the order in which the different stages of the development occur. The best classification performance on these types of images was found with our optimized CNN, which achieved 94% accuracy of detection. In our experiments all models and fusion strategies were trained and tested on several genotypes of beans.

## Figures and Tables

**Table 1:** Description of the RGB-Depth dataset used in this study.

	Species	No. of Temporal Sequences	Totale No. of Images during Days	Totale No. of Images during Nights	Totale No. of All Images
Training dataset	Flavert	10	4240	1920	36,960
	Red Hawk	10	4240	1920	
	Linex	10	4240	1920	
	Caprice	10	4240	1920	
	Deezer	10	4240	1920	
	Vanilla	10	4240	1920	
Validation dataset	Flavert	1	424	192	3696
	Red Hawk	1	424	192	
	Linex	1	424	192	
	Caprice	1	424	192	
	Deezer	1	424	192	
	Vanilla	1	424	192	
Testing dataset	Flavert	1	424	192	3696
	Red Hawk	1	424	192	
	Linex	1	424	192	
	Caprice	1	424	192	
	Deezer	1	424	192	
	Vanilla	1	424	192	

**Table 2:** Seedling growth stage classification average accuracy and standard deviation when performed over 10 repetitions of CNN model.

	Training	Validation	Test
RGB	0.95±0.02	0.91±0.03	0.88±0.05
Image fusion RGB-Depth	0.97±0.02	0.95±0.02	0.94±0.04
Features fusion RGB-Depth	0.97±0.01	0.96±0.01	0.94±0.01

**Table 3:** Seedling growth stage classification average accuracy and standard deviation when performed over 10 repetitions of TD-CNN-GRU model.

	Training	Validation	Test
RGB	0.87±0.02	0.85±0.01	0.80±0.01
Image fusion RGB-Depth	0.91±0.01	0.87±0.02	0.82±0.01
Features fusion RGB-Depth	0.90±0.01	0.86±0.02	0.81±0.01

**Table 4:** Seedling growth stage classification average accuracy and standard deviation when performed over 10 repetitions of transformer model.

	Training	Validation	Test
RGB	0.90±0.02	0.86±0.01	0.82±0.01
Image fusion RGB-Depth	0.96±0.02	0.91±0.01	0.88±0.03
Features fusion RGB-Depth	0.92±0.03	0.89±0.02	0.84±0.01

**Table 5:** Training time of the different deep learning architectures.

	Model	Training Time
RGB	CNN	1 h 00 min
	Transformer	1 h 30 min
	TD-CNN-GRU	3 h 00 min
Image fusion RGB-Depth	CNN	1 h 15 min
	Transformer	1 h 35 min
	TD-CNN-GRU	3 h 30 min
Features fusion RGB-Depth	CNN	1 h 20 min
	Transformer	1 h 30 min
	TD-CNN-GRU	3 h 20 min

## Data Availability

Data are available upon reasonable request.
